# The tumor–microbe connection

**DOI:** 10.1002/1878-0261.70115

**Published:** 2025-09-03

**Authors:** Gerlanda Vella, Maria Rescigno

**Affiliations:** ^1^ IRCCS Humanitas Research Hospital Milan Italy; ^2^ Department of Biomedical Sciences Humanitas University Milan Italy

**Keywords:** intratumoral microbiota, metastasis, therapy efficacy, tumor microenvironment

## Abstract

The discovery of tumor‐associated bacteria (TAB) challenges the traditional view of tumors as sterile environments. These microbes are engaged in a complex dialog with the other components of the tumor microenvironment (TME), influencing immunity, metastasis, and treatment response. Yet the precise mechanisms by which TAB influence tumor biology remains incompletely understood. Deciphering the complex host–microbe interactions could unlock novel therapeutic strategies to reshape the TME and improve treatment outcomes. Here we summarize the key findings in the field, highlighting the most outstanding questions regarding bacterial sources, the roles of TAB in cancer, and their interactions with the other cellular components of the TME.

## Graphical abstract

Tumor‐associated bacteria (TAB) dynamically influence cancer biology by modulating tumor progression, metastatic spread, and therapeutic efficacy. Their presence redefines the tumor microenvironment (TME) as a microbial–host interface, yet mechanistic insights remain limited. Understanding TAB colonization routes, functional impacts, and crosstalk with other cellular components of the TME may pioneer novel bacteria‐targeted cancer therapies.

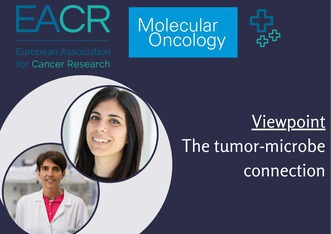

**Abbreviations**
5‐FU: 5‐fluorouracil
AhR: Aryl hydrocarbon receptor
B. fragilis: *Bacteroides fragilis*
CagA: Cytotoxin‐associated gene A
CDH1: Cadherin 1
CRC: Colorectal cancer
CREB: cAMP‐response element binding protein
*E. coli*
: *Escherichia coli*
EMT: Epithelial‐to‐mesenchymal transition
ETBF: Enterotoxigenic *Bacteroides fragilis*
*F. nucleatum*
: *Fusobacterium nucleatum*
FadA: *Fusobacterium* adhesin A
FAP: Familiar adenomatous polyposis
Fap2: Fibroblast activation protein 2
GalNAc: N‐Acetylgalactosamine
GC: Gastric cancer
*H. pylori*
: *Helicobacter pylori*
I3A: Indole‐3‐aldehyde
IFNγ: Interferon‐γ
*L. iners*
: *Lactobacillus iners*
*L. paracasei*
: *Lactobacillus paracasei*
*L. reuteri*
: *Lactobacillus reuteri*
MHC‐II: Major histocompatibility complex class‐II
MSS: Microsatellite‐stable
NGS: Next‐generation sequencing
Pks: Polyketide synthetase
*S. anginosus*
: *Streptococcus anginosus*
TAB: Tumor‐associated bacteria
TBX21: T‐box transcription factor 21
TME: Tumor microenvironment

## 1 Introduction

For over a century, microorganisms have been detected within tumors, yet their presence was long met with skepticism. Only recently, thanks to advances in next‐generation sequencing (NGS), the intratumoral microbes are recognized as integral components of the tumor microenvironment (TME). The discovery of diverse microbiota—bacteria, viruses, fungi, and protozoa—coexisting within tumors has challenged the traditional view of tumors as sterile entities. We now appreciate tumors as complex, dynamic ecosystems in which microbial and human cells coexist and interact. This revelation raises important questions: What exactly are tumor‐associated bacteria doing in the TME? Are they merely passive bystanders, exploiting the tumor's resources, or are they active participants—shaping tumor progression, modulating immune responses, or even influencing therapy outcomes? Could certain microbes accelerate malignancy, while others suppress it? And if so, might targeting these microbial communities open new avenues for cancer treatment? Addressing these questions is essential to fully elucidate the role of the tumor‐associated microbiota in cancer biology.

## 2 Routes of bacterial colonization

The tumor mass, with its typical immunosuppressed, nutrient‐rich, and hypoxic conditions represents a favorable environment for bacteria colonization and persistence, particularly for anaerobic species. Moreover, microbial adaptations, including phagocytosis resistance and biofilm formation, further support their survival within tumors. Rather than being evenly distributed, bacteria tend to localize within poorly vascularized microniches enriched with protumoral macrophages, highlighting the importance of the local microenvironment within the tumor mass [1]. But where do these bacteria come from? Well, a univocal answer to this question doesn't exist, as three putative routes of bacteria dissemination have been proposed.

The bloodstream likely serves as the primary highway for bacterial entry to tumors. Bacteria may exploit tumor‐specific features for tumor colonization. The characteristic vascular permeability of tumors may facilitate passive bacterial entry, while specific molecular interactions, such as the binding of bacterial Fap2 to host GalNAc, enable active targeting of malignant tissues [2].

Alternative routes comprise mucosal barriers, particularly relevant for tumors at epithelial surfaces like colorectal cancer (CRC) or lung cancers, and the adjacent healthy tissues. Regarding this last route, there are two possibilities: either microbes migrate from healthy tissue into developing tumors, or local microbial communities residing in healthy tissue expand as the tumor forms. The directionality of this relationship remains unclear, as does the fundamental question of how bacteria normally colonize healthy internal organs. Notably, some species employ multiple colonization strategies. For instance, *Fusobacterium nucleatum*, an oral commensal, abundantly found in human CRC, can reach tumors via both the direct migration through the gastrointestinal tract and the hematogenous spread during transient bacteremia occurring during daily oral hygiene [3, 4].

## 3 Tumor establishment

The observation that certain bacterial species are more abundant in malignant tissues compared to adjacent healthy tissues—especially in advanced stage tumors—suggests that these microbes may play an active role in carcinogenesis and tumor progression. Probably one of the most prominent examples is *F. nucleatum*. The ability of this bacterium to influence cancer progression is largely mediated by its adhesin protein, FadA, which binds to CDH1 on the surface of host epithelial cells and activates the protumorigenic Wnt/β‐catenin signaling pathway [5]. Other examples include *Helicobacter pylori* and *Streptococcus anginosus*, both implicated in gastric cancer (GC) carcinogenesis. *H. pylori* translocates the CagA oncoprotein into gastric epithelial cells, triggering proinflammatory signaling pathways, while *S. anginosus* directly interacts with gastric epithelial cells and activates the MAPK pathway, promoting tumorigenesis [6, 7]. Similarly, enterotoxigenic *Bacteroides fragilis* (ETBF), particularly enriched in precancerous lesions in familiar adenomatous polyposis (FAP) patients, drives colonic tumorigenesis by activating IL‐17‐dependent NF‐κB signaling and inducing chronic inflammation [8].

**Fig. 1:**
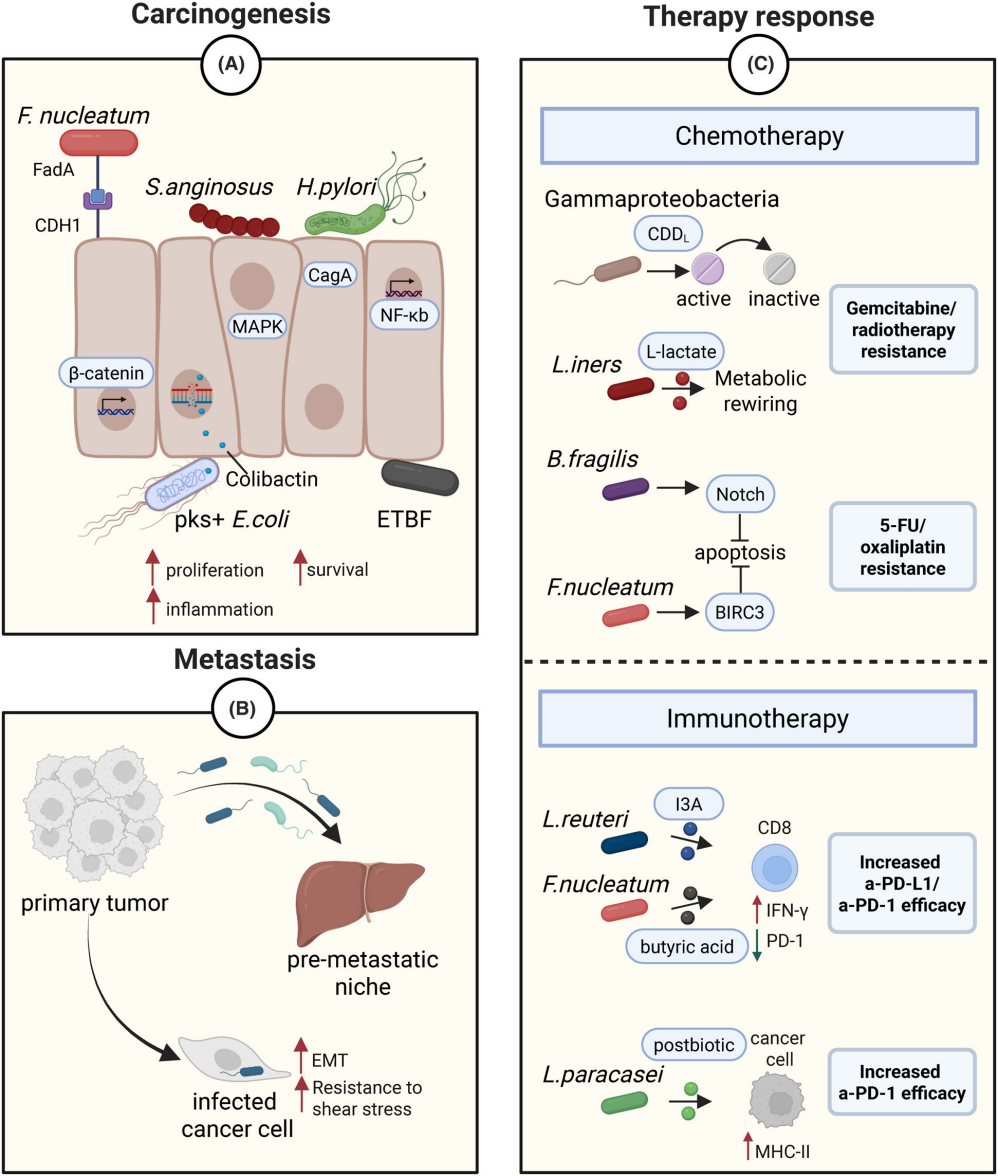
Bacterial modulation of cancer biology. (A) Certain bacteria can promote carcinogenesis by favoring cell proliferation/survival and promoting inflammation: *F. nucleatum* activates the Wnt/β‐catenin signaling pathway upon binding to CDH1 on the surface of host epithelial cells via the adhesin protein FadA; *H. pylori* triggers proinflammatory signaling pathways after translocating its CagA oncoprotein into gastric epithelial cells; *S. anginosus* activates the MAPK pathway; ETBF activates IL‐17‐dependent NF‐κB signaling; pks + *E. coli* induces DNA damage via its genotoxic colibactin metabolite. (B) Bacteria can promote metastasis by favoring the establishment of a premetastatic niche, inducing the EMT in cancer cells, and enhancing the shear stress resistance via host cytoskeletal remodeling. (C) Tumor‐associated bacteria can influence the response to chemotherapy and immunotherapy: Gammaproteobacteria inactivate gemcitabine via the cytidine deaminase (CDDL); *B. fragilis* promotes 5‐FU/oxaliplatin resistance through Notch activation and apoptosis inhibition; *F. nucleatum* induces 5‐FU resistance via the antiapoptotic factor BIRC3 upregulation; *L. iners* confers radiation/gemcitabine resistance via L‐lactate‐mediated metabolic reprogramming; *L. reuteri* enhances anti‐PD‐L1 efficacy via AhR‐mediated IFNγ production in CD8^+^ T cells; *F. nucleatum*‐derived butyrate and *L. paracasei*‐derived postbiotic improve anti‐PD‐1 response via PD‐1 downregulation in CD8^+^ T cells and MHC‐II upregulation in cancer cells, respectively.

Some bacteria can directly cause DNA damage. For instance, certain *Escherichia coli* strains carry the polyketide synthase (pks) pathogenicity island, encoding enzymes that synthesize colibactin. This toxin induces DNA adducts and double‐strand breaks, contributing to genomic instability in CRC [9] (Fig. 1A).

Collectively, these findings suggest that bacteria may play a broader and more dynamic role in cancer development than previously appreciated. Unraveling the intricate crosstalk between tumor‐associated bacteria (TAB) and the host could reveal novel mechanisms of tumorigenesis and potentially uncover innovative strategies for cancer prevention and therapy.

## 4 Metastases

Either freely circulating in the bloodstream or residing within circulating cancer cells, bacteria can alter the metastatic process through three main mechanisms: (1) counteracting fluid shear stress via host cytoskeleton remodeling [10]; (2) creating a premetastatic niche [11]; (3) modulating the host epithelial‐to‐mesenchymal transition (EMT) [12] (Fig. 1B).

The idea that the tumor cell spread is not random dates back to the late 19th century with Paget's “seed and soil” hypothesis, where cancer cells (“seeds”) grow only in favorable organ environments (“soil”). Recent research expands this view, showing that bacterial communities in metastases are influenced more by the metastatic site than the primary tumor type [13]. This suggests the intriguing possibility of organ‐specific microbial tropisms, where certain bacteria are drawn by tissue factors like nutrients or signals, shaping a microenvironment that supports metastasis. Given its potential significance, this idea warrants further investigation to clarify the role of microbes in metastasis and how they might be targeted in cancer treatment.

## 5 Therapy response

When we think of therapy resistance in cancer, one of the first concepts that comes to mind is the innate or acquired resistance driven by genetic mutations within cancer cells. However, the mechanisms of drug resistance extend beyond tumor‐intrinsic genetic alterations and can also be influenced by TAB (Fig. 1C). This can occur by direct modification of the drugs. This is the case of Gammaproteobacteria that can convert the gemcitabine into its inactive form via the bacterial cytidine deaminase, limiting gemcitabine's therapeutic efficacy in a murine CRC model [14]. Alternatively, bacteria‐driven chemoresistance can rely on the host metabolic and cellular pathway alterations. *Bacteroides fragilis*, which is more abundant in chemoresistant than in responder CRC, promotes resistance to 5‐fluorouracil (5‐FU) and oxaliplatin, through the surface protein SusD/RagB that interacts with and activates the Notch1 prosurvival signaling pathway in host cells. Consistently, elimination of *B. fragilis* using targeted phage therapy successfully restored chemosensitivity in CRC mouse models [15]. In cervical tumors, *Lactobacillus iners* appears to contributes to resistance against both gemcitabine and radiation. It has been proposed that this effect could be mediated by bacterial L‐lactate, which reprograms cancer cells to rely on lactate metabolism, thereby potentiating lactate utilization over glucose under stress conditions, including irradiation [16].

In addition to regulating the efficacy of antitumor drugs, TAB can profoundly impact immunotherapy outcomes by modulating the host's immune system. For instance, *Lactobacillus reuteri* migrates to, colonizes, and persists within murine melanoma tissue, where it promotes antitumor immunity and enhances the efficacy of anti‐PD‐L1 therapy. This effect occurs through activation of the AhR in CD8^+^ T cells by the bacterial metabolite I3A, which leads to IFNγ production in a CREB‐dependent manner [17]. Moreover, a *Lactobacillus paracasei*‐derived postbiotic upregulates MHC‐II expression on murine breast cancer cells, boosting anti‐PD‐1 immunotherapy efficacy [18]. Notably, the same bacterium can exert opposing effects. For instance, *F. nucleatum* contributes to CRC carcinogenesis and impairs 5‐FU efficacy by upregulating the host's antiapoptotic factor BIRC3 [19], yet it may also sensitize microsatellite‐stable (MSS) CRC tumors to anti‐PD‐1 immunotherapy. This is mediated by the bacterial butyric acid, which enhances CD8^+^ T cell activity through TBX21‐mediated PD‐1 downregulation. Consistently, high intratumoral *F. nucleatum* levels correlate with favorable responses, suggesting its potential as a biomarker for immunotherapy in MSS CRC [20]. In light of these observations, the TAB should be considered a potential stratification factor when selecting anticancer therapies. Screening for specific microbial signatures may enhance the ability to predict treatment outcomes and guide personalized therapeutic strategies. An additional layer of complexity arises from the variability in bacterial load across different tumor types. While the presence of certain microbes is clearly linked to therapy resistance or efficacy in tumors with high bacterial load, such as melanoma and CRC, the extent to which this effect is relevant in tumors harboring low bacterial loads remains uncertain. It is plausible that a threshold level of microbial burden is required to significantly influence therapy response.

## 6 Concluding remarks

Growing appreciation is being given to the TAB within the TME, as they can play a dual role—either promoting tumor growth and therapy resistance or supporting antitumor immunity. Targeted strategies to eliminate harmful TAB, such as using antibiotics or bacteriophages, while preserving beneficial bacteria, are therefore essential. Despite advances, several key questions remain unresolved: What evolutionary pressures enable certain bacteria to adapt to the tumor niche? How do TAB interact causally and spatially with other TME components, like immune and stromal cells? Do specific bacteria show preference for certain metastatic sites, and what governs this tropism? Moreover, can microbial signatures within tumors serve as reliable biomarkers for prognosis or treatment response? Alongside these, the dynamic interactions within bacterial communities themselves—whether competitive or cooperative—must also be considered. Ultimately, understanding the complex roles and interactions of TAB in the TME may reveal novel molecular targets for innovative anticancer therapies.

## Conflict of interest

MR is the founder and CSO of Postbiotica srl.

## Author contributions

GV conducted the literature review and wrote the article. MR provided expert feedback, revised the article, and approved the final version.
